# Knowledge Retrieval from PubMed Abstracts and Electronic Medical Records with the Multiple Sclerosis Ontology

**DOI:** 10.1371/journal.pone.0116718

**Published:** 2015-02-09

**Authors:** Ashutosh Malhotra, Michaela Gündel, Abdul Mateen Rajput, Heinz-Theodor Mevissen, Albert Saiz, Xavier Pastor, Raimundo Lozano-Rubi, Elena H. Martinez-Lapsicina, Irati Zubizarreta, Bernd Mueller, Ekaterina Kotelnikova, Luca Toldo, Martin Hofmann-Apitius, Pablo Villoslada

**Affiliations:** 1 Department of Bioinformatics, Fraunhofer Institute for Algorithms and Scientific Computing, 53754, Sankt Augustin, Germany; 2 Merck KGaA, Darmstadt, Germany; 3 MS Center, Department of Neurology, Hospital Clinic of Barcelona, Barcelona, Spain; 4 Center of Neuroimmunology, Institut d'investigacions Biomèdiques August Pi i Sunyer (IDIBAPS), Barcelona, Spain; 5 Department of Medical Informatics, Hospital Clinic of Barcelona—University of Barcelona, Barcelona, Spain; Mathematical Institute, HUNGARY

## Abstract

**Background:**

In order to retrieve useful information from scientific literature and electronic medical records (EMR) we developed an ontology specific for Multiple Sclerosis (MS).

**Methods:**

The MS Ontology was created using scientific literature and expert review under the Protégé OWL environment. We developed a dictionary with semantic synonyms and translations to different languages for mining EMR. The MS Ontology was integrated with other ontologies and dictionaries (diseases/comorbidities, gene/protein, pathways, drug) into the text-mining tool SCAIView. We analyzed the EMRs from 624 patients with MS using the MS ontology dictionary in order to identify drug usage and comorbidities in MS. Testing competency questions and functional evaluation using F statistics further validated the usefulness of MS ontology.

**Results:**

Validation of the lexicalized ontology by means of named entity recognition-based methods showed an adequate performance (F score = 0.73). The MS Ontology retrieved 80% of the genes associated with MS from scientific abstracts and identified additional pathways targeted by approved disease-modifying drugs (e.g. apoptosis pathways associated with mitoxantrone, rituximab and fingolimod). The analysis of the EMR from patients with MS identified current usage of disease modifying drugs and symptomatic therapy as well as comorbidities, which are in agreement with recent reports.

**Conclusion:**

The MS Ontology provides a semantic framework that is able to automatically extract information from both scientific literature and EMR from patients with MS, revealing new pathogenesis insights as well as new clinical information.

## Introduction

To understand MS it is necessary to integrate information from several different sources using advanced computational tools [1–3]. However, the first challenge to be met is to retrieve useful information from the multiple sources available (structured databases, narrative text in scientific articles, medical information in clinical notes) despite the different data standards and qualities. Currently, a tremendous amount of information is available through the scientific literature (e.g. 62,364 articles on MS at PubMed by October 2014), a number that is steadily increasing. Information retrieval is not the creation of new knowledge and for this reason it is necessary to use specific tools to exploit this vast quantity of data. For this reason, the use of automated systems to retrieve information, which will scan scientific literature sources on the basis of medical concepts, has gained much attention in the field of medical informatics, leading to the development of dedicated text-mining systems.

One-way of retrieving information from these complex sources is to use ontologies and text-mining tools. In medical informatics, Ontology is a computational tool that represents knowledge as a set of concepts (words) within a domain (e.g. MS), using a shared vocabulary (dictionary) to denote the types, properties and interrelationships between such concepts (symptoms, drugs, molecules, pathways, etc.) [4]. Ontologies have been used extensively to retrieve and integrate biological information (e.g. Gene Ontology), or medical information, such as the Alzheimer's disease ontology, that enabled us to obtain additional information from PubMed abstracts and electronic medical records (EMR) (e.g. identifying hypertension, diabetes and stroke as the most common co-morbidities for AD) [5].

In this study we aimed to develop an ontology specific for MS for clinical and translational research. Also, we envisage that in the near future they can be used at the clinical level to retrieve information from EMRs in order to design more tailored healthcare for given populations.

## Methods

### Ethical Statement

This study was approved by the Ethical Committee of the Hospital Clinic of Barcelona, which provided a waiver for the request of the patients’ written informed consent. All clinical investigation have been conducted according to the principles expressed in the Declaration of Helsinki

### Electronic Health Records from patients with MS

We analyzed the EMRs of MS patients from the Hospital Clinic of Barcelona. The EMR system at our center is at level 6 of the HIMSS category (http://www.himss.org/) since 2011. MS cases were retrieved from the database of the MS center, or by using the ICD-9 code 340, or the key words “Multiple Sclerosis” or “demyelinating disease” in the free text of the medical notes. We identified 734 records from patients fulfilling this search criteria. Diagnosis was confirmed by a specialized neurologist (PV), making 624 MS cases available for analysis. Patients were excluded mainly because MS was cited as part of the differential diagnosis but the disease was not confirmed. We also noted that the diagnosis of “Systemic Sclerosis” was included in the results and thus, this diagnosis was introduced into subsequent searches as a specific exclusion criteria. We collected all the notes from any physician who has ever participated in the patient’s care, not only those of the neurologists, as well as discharge letters or emergency room letters, and exported them as anonymized pdf files for further analysis. This study was approved by the IRB of the Hospital Clinic of Barcelona, which provided a waiver for the request of the patients’ written informed consent.

### Development of the MS ontology

The MS Ontology was constructed using the same approach as the AD ontology described previously [5]. Briefly, we used the Protege OWL editor (version 4.2; http://protege.stanford.edu) to build the MS Ontology. A collection of terms and concepts related to MS were generated by scanning various knowledge sources, such as scientific articles, the content of online books, medical knowledge bases, encyclopedias, glossaries, and online information sources and websites. We developed a dictionary in English with concepts, definitions and synonyms, and then translated it to Spanish and Catalan to analyze the EMRs (S1 Table). Classes were annotated with synonyms, both manually and in an automated way, making use of mappings to external ontologies provided by the National Center for Biomedical Ontology [6]. For entity (word) recognition within a text, the dictionary was incorporated into the ProMiner software [7]. The ontology is freely available at Bioportal (http://purl.bioontology.org/ontology/MSO) and at the Fraunhofer website: http://www.scai.fraunhofer.de/de/geschaeftsfelder/bioinformatik/downloads.html

**Figure 1:**
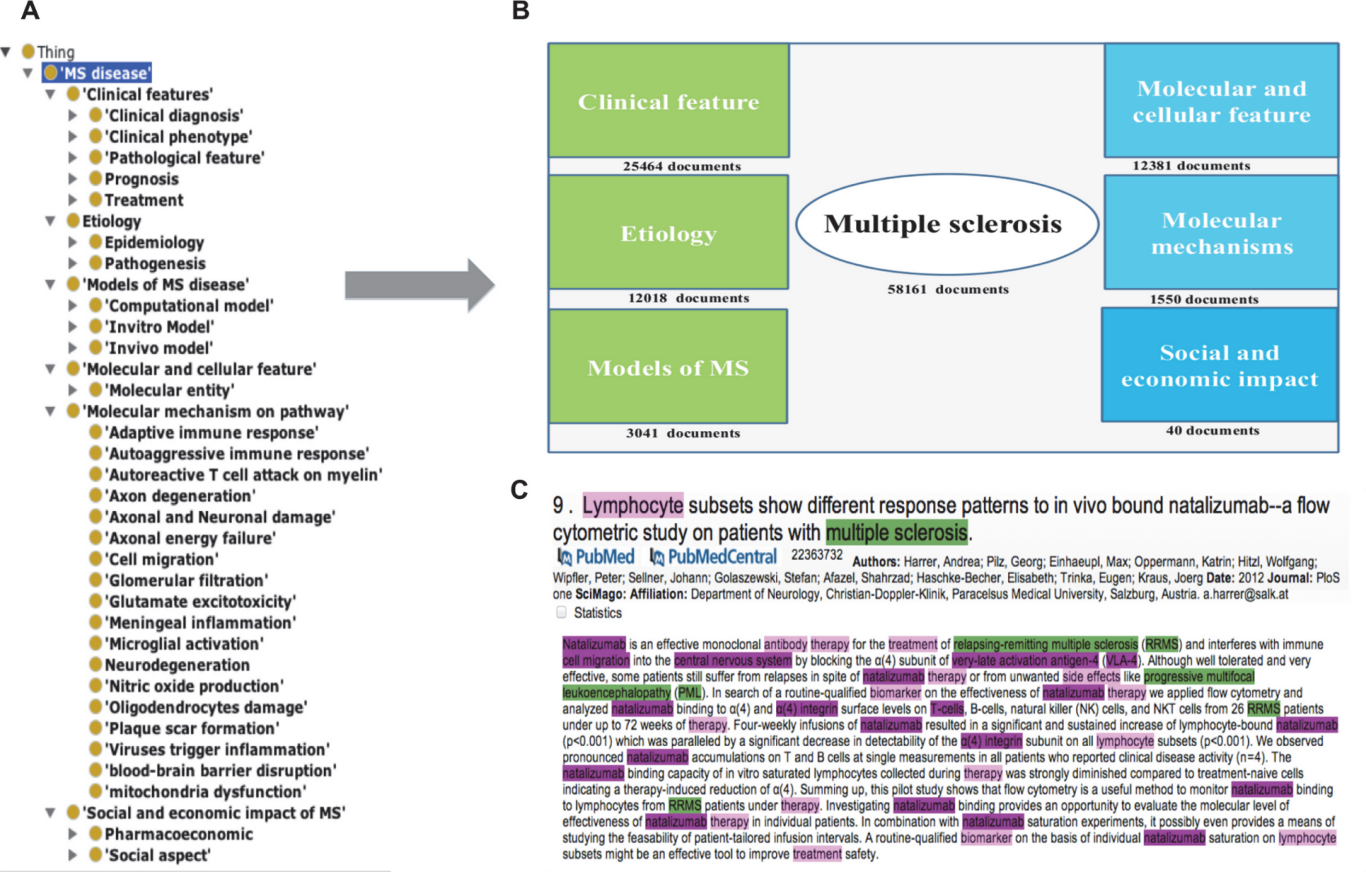
The MS Ontology. A) Basic formal ontology integration of MS Ontology; B) Extracted views of the MS Ontology showing the hierarchy of the concepts; C) Source documents for each category used for creating the ontology.

In a subsequent step, various “class-concepts” were used as keywords to search PubMed abstracts in order to build a corpus that covers the MS domain: MS biomarkers, brain regions, diagnostic procedures, therapies, epidemiology, etiology, genetics, pathogenesis, stages, symptoms, clinical trials, and risk factors (Fig. 1). For enrichment purposes, the training set was analyzed for false-negative entities that were added to the MS Ontology terminology after individual expert evaluation. Moreover, MS experts cross-checked the whole ontology and additional expert knowledge was incorporated. See S1 Methods for details.

### Analysis of MS concepts in PubMed abstracts and EMRs

To retrieve and analyze concepts from PubMed abstracts, the MS Ontology was integrated into the SCAIView text-mining system (http://www.scaiview.com). SCAIView is a text-mining software that is able to identify terms and connect them using dictionaries. The MS Ontology dictionary is available: 1) as a hierarchy tree; 2) as a searchable tool using auto-completion; 3) by highlighting results in documents (pdf); and 4) showing results in the “Entity View”. In order to analyze EMRs, we linked the MS Ontology dictionary to the DrugBank dictionary to retrieve drug usage information and to the Medical Subject Heading (MeSH) terms to identify co-morbidities. The performance of the MS Ontology in comparison to manual searches in PubMed was analyzed using F statistics as described elsewhere [5].

## Results

### Development and evaluation of the MS Ontology

The MS Ontology was developed using the standards from the National Center for Biomedical Ontology and represent medical knowledge specific to MS. The MS Ontology used a hierarchy of concepts in the MS knowledge domain, including: 1) Clinical features, 2) Etiology, 3) Models of MS, 4) Molecular mechanisms on pathways, 5) Molecular and cellular features, and 6) Social and economic impact of MS (Fig. 1, see S1 Methods for details). We evaluated the MS Ontology on the abstract test set, founding that the MS Ontology could automatically retrieve a wide range of MS concepts (F = 0.73, see the example in S1 Fig.). The expert panel’s revision is considered to be a genuine evaluation for disease ontologies [8], and allowing this revision by experts in MS, we curated the ontology manually.

We designed three clinical or scientific queries (competency questions) that were defined by the experts to evaluate the performance of the MS Ontology in returning appropriate information regarding disease pathogenesis (question 1 and 2) and therapies (question 3). The questions were designed in order to relate at least 3 different but common concepts, a kind of search strategy that manual PubMed searches use to provide few results (false negative) or non-specific/inaccurate results (false positive) (see S1 Methods for details):

1. Return references linking brain atrophy and remyelination and MS.
2. Return references linking Myelin Oligodendrocyte Glycoprotein and antibody-mediated demyelination and MS.
3. Return references linking fingolimod and phase 3 clinical trials and RRMS.

**Figure 2:**
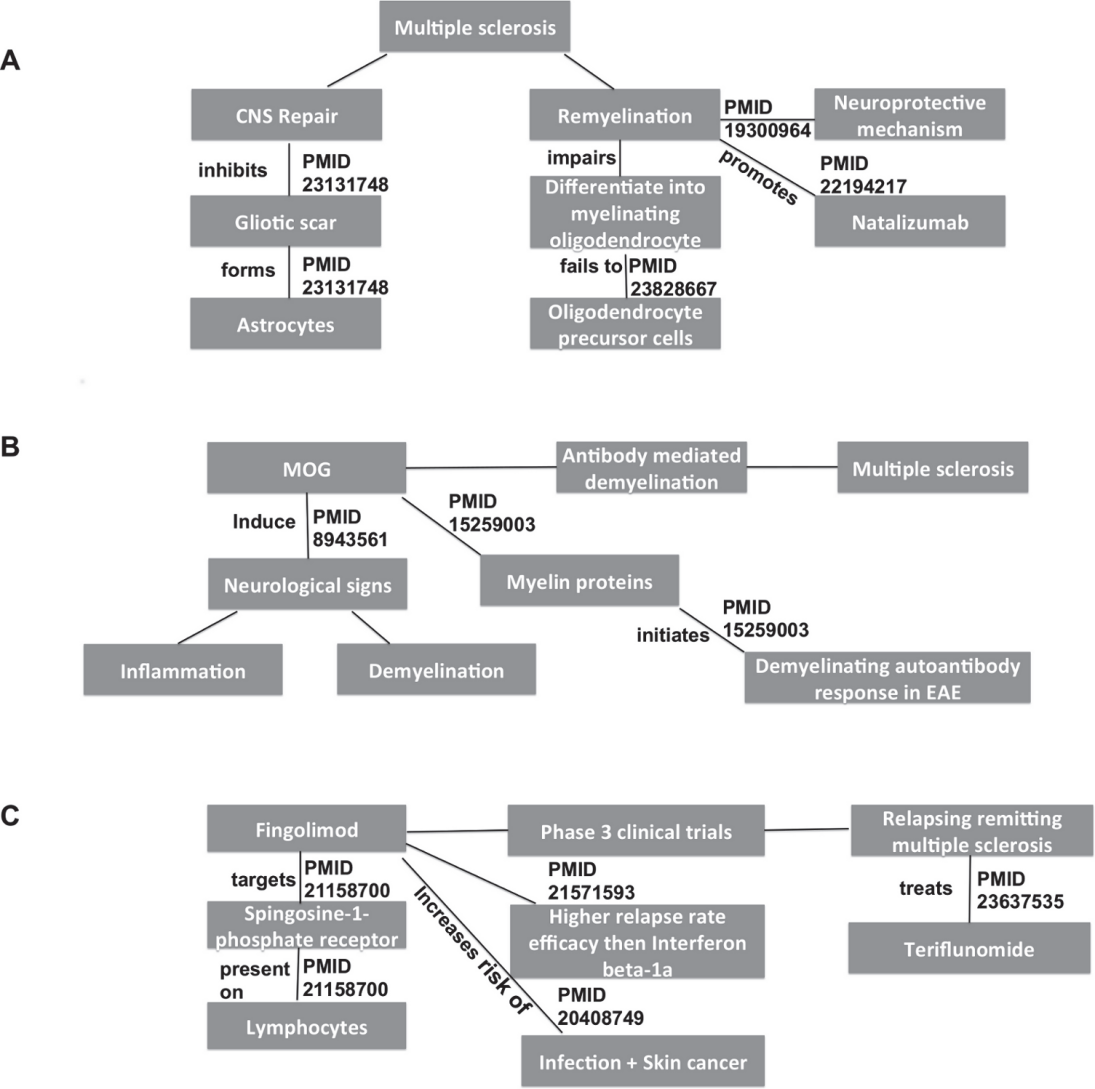
Concepts identified using the MS Ontology in the competency questions. Figure shows the concepts (in grey boxes) retrieved in the competency questions (search strategy) annotated by the MS Ontology and linked to other MS Ontology concepts, indicating the PMID of the abstract from PubMed and the type of interaction described in such abstract. A) references linking brain atrophy and CNS repair with remyelination in MS; B) references linking Myelin Oligodendrocyte Glycoprotein (MOG) to antibody-mediated demyelination; and C) references linking fingolimod tested as a drug for treatment of relapsing-remitting MS in phase 3 clinical trials

To evaluate the queries, we compared documents returned by the MS Ontology with documents returned by “advance search” in PubMed (we used manual search in PubMed using keywords and revised by an expert as a gold standard). We found that the MS Ontology obtained a lower ratio of false positive and false negative results than manual searching (Table 1). Nevertheless, the information retrieved from PubMed with the MS Ontology provided a structure on the basis of the relationship between terms, allowing the hierarchy and logic of the evidence found in scientific abstracts to be followed (Fig. 2). These results indicate that MS Ontology-based information retrieval improved the chances of gaining more accurate (decreasing false positive and negative results) and structured information compared to PubMed advance searches.

**Table 1:** Results of competency questions evaluation using MS Ontology compared to manual search on PubMed.

Question No.	1	2	3
# Documents retrieved by MS Ontology	26	9	27
• Validated documents (MS Ontology)	26	9	26
Specificity of MS Ontology	100%	100%	96%
# Documents retrieved by PubMed advanced search	0	3	1
• Validated documents (PubMed advance search)	0	2	1
# Documents retrieved by expert search in PubMed	18	11	14
• Validated documents (expert search)	15	9	12
• Sensitivity of MS Ontology	100%	100%	100%

Results are shown as the number of all retrieved documents and the “validated ones” based in manual review of the documents by the expert in order to ensure they were covering the topics of the competency questions. We define as the gold standard for calculating sensitivity, the expert search in PubMed using key words (related with AND) and the manual revision of the abstracts. In order to calculate ‘Sensitivity’ and ‘Specificity’ of MS Ontology based searches, true positives are defined as the number of ‘validated documents’ retrieved by a MS Ontology based search; false positive are the number of documents retrieved by MS Ontology based search but were not considered relevant in expert review and False negatives are the number of documents retrieved by ‘expert based searches’ in PubMed but were not retrieved by MS Ontology. See S1 Methods for details of the searches.

### Mining PubMed abstracts using MS Ontology

To validate the use of the MS Ontology as an automatic tool to obtain scientific information from PubMed abstracts, we combined the MS Ontology dictionary with dictionaries of human genes, proteins and pathway. To explore the association between MS and genetic predisposition, we retrieved the list of genes associated with MS susceptibility using the MS Ontology and compared this list with the genes validated in GWAS studies identified by the expert search [9, 10]. Through the automatic search of PubMed with the MS Ontology we retrieved up to 80% of the genes mentioned in such GWAS studies (S2 Table). Moreover, using the MS Ontology we retrieved 13 genes (TMEM39A, ERAP1, KIF5A, DHCR7, CD226, TYK2, DEXI, MYTIL, ZFP57, C7, SCIN, DPP6, PSMB9) that have been associated with MS in GWAS studies [13, 14] but that were not mentioned even in the main text of the article but rather in other sections of the articles (e.g. tables, supplementary material, etc.). Therefore, the MS Ontology was useful as an automated system to retrieve highly specific scientific information from a wide knowledge area (e.g. genes associated with autoimmune diseases) without manual supervision.

**Figure 3:**
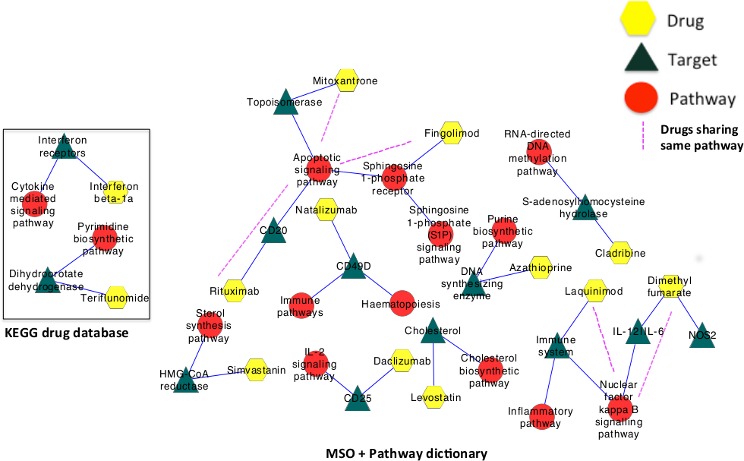
Drug-target-pathway map of MS drugs using MS Ontology compared to KEGG database. A search in the KEGG database (a database of molecular pathways and drugs), identified pathways associated with interferon-beta and teriflunomide (box in the left). The automatic retrieval using MS Ontology identified additional pathways for all current MS disease modifying therapies, including mitoxantrone, natalizumab, azathioprine, laquinimod, simvastatin, levostatin, dimethyl-fumarate, rituximab and daclizumab and their interactions.

As a second example, we used the MS Ontology in combination with the pathway dictionary to analyze PubMed abstracts in order to generate maps that relate current disease-modifying drugs (DMD) for MS to their molecular targets (receptors) and downstream pathways. We compared our results with a search in the KEGG database (http://www.genome.jp) as the reference database for biological pathways. We found that KEGG only contains drug-target-pathway information for inteferon-beta1a and teriflunomide, whereas the MS Ontology identified additional drug-target-pathway maps for MS therapies (Fig. 3). Such new drug-pathway maps will require experimental validation and can be used as starting hypothesis for the analysis of the biological effects of drugs in MS.

### Automatic analysis of electronic medical records from MS patients using MS Ontology

In order to assess the usefulness of MS Ontology to extract information from the EMRs of patients with MS, we used MS Ontology with the English dictionaries translated into Spanish and Catalan, to mine the EMR of 624 MS patients. We focused on retrieving information regarding the presence of co-morbidities and the use of drugs because these topics have been analyzed by recent studies and will serve as reference [11–23]. Second, because even if this information can be obtained straightforward from the EMR, the MS Ontology should be able to relate with other concepts in MS. An example of two anonymized medical records on which the search for drug usage and co-morbidities is shown in S2 Fig. Regarding drug usage by MS patients, we found the frequency of DMD use was consistent with recent surveys in Europe, US and Canada (Table 2, S3 Table) [11–15]. In terms of the use of symptomatic therapy, we observed a similar high use of analgesics and benzodiazepines as it has been described previously [16–19]. We also analyzed the presence of co-morbidities in the EMRs of MS patients using the MS Ontology. Accordingly, we found a high frequency of CNS/psychiatric, cancer, cardiovascular, or metabolic diagnoses, in accordance with previous studies using other approaches, such as self-registries, databases, etc. (Table 3, S4 Table) [20–23]. Overall, the MS Ontology was able to retrieve information from the EMR regarding drug usage and comorbidities, which is in agreement with recent surveys in the field. Such information can be related with other clinical and biological concepts with the use of the MS Ontology in order to generate new hypothesis for future clinical research.

**Table 2:** Top 5-drug usage by patients with MS identified in the EMR.

No	Disease modifying therapies	%
1	Interferon-beta	43%
2	Glatiramer Acetate	17%
3	Natalizumab	7%
4	Fingolimod	7%
5	Rituximab	0.5%

No	Symptomatic therapy	%
1	Acetaminophen	18%
2	Aminosalicylic Acid	12%
3	Baclofen	9%
4	Lorazepam	5%
5	Diazepam	5%

**Table 3:** Comorbidities diagnosed in patients with MS identified in the EMR.

No	Disease class	%
1	Nervous system diseases	31%
2	Neoplasms	14%
3	Musculoskeletal disorders	13%
4	Otorhinolaryngologic Diseases	10%
5	Eye diseases	7%
6	Mental disorder	7%
7	Eye disease	7%
8	Congenital, hereditary and neonatal diseases and abnormalities	5%
9	Cardiovascular diseases	5%
10	Immune system diseases	5%
11	Nutritional and metabolic disorders	5%
12	Respiratory tract diseases	4%
13	Skin and connective tissue diseases	4%
14	Female urogenital diseases	4%
15	Endocrine system diseases	4%
16	Digestive system diseases	4%
17	Bacterial infections and Mycoses	4%
18	Behavior and behavioral mechanisms	3%
19	Male urogenital diseases	3%
20	Viral diseases	2%
21	Hemic and lymphatic diseases	2%
22	Parasitic diseases	0.6%

## Discussion

In this study we describe a new tool for automatic information retrieval in MS by developing an ontology specific for MS. Applying an ontology-driven information mining approach that models MS related concepts and hierarchies, enhances the quality of information searches. At present, PubMed is the only "open access" engine available to search for MS related information. Although, the search capacity of PubMed has increased tremendously in the last decade (e.g advance search options), there is still a considerable effort demanded of users to obtain the information in which they are interested in (e.g. search using "keywords" and manual review of abstracts). By contrast, MS Ontology has the ability to answer all such queries. Additionally, further information is obtained by linking the MS Ontology to other specific terminologies within the text-mining software, such as other disease ontologies, drug dictionaries, pathway information, or gene and protein dictionaries.

The applications presented here demonstrate some potential uses of the MS Ontology for translational and clinical research in the field of MS. MS Ontology performed adequately when compared with reference search engines (advanced searches of PubMed or of searches in the KEGG database for pathway analysis). In addition, its application to the analysis of EMR exemplifies how text-mining can be performed with MS Ontology. Therefore, at present the MS Ontology can only be used by MS researchers in studies that wish to exploit the exponential growth in scientific literature that cannot be approached by manual searches in PubMed, and the almost unexplored information contained in the EMRs (e.g Phenome wide association studies (PheWAS)) [24]. We also envisage that this tool will be useful for clinicians interested in analyzing health care strategies for their specific population of MS patients.

In recent years, the benefits of ontologies have become evident in data management, integration and processing, in both the biological and medical domain. In the case of AD [5], the development of a specific ontology was critical because the amount of research conducted every year is significantly higher than that of other diseases such as MS, epilepsy or Parkinson disease. Moreover, the epidemiology of dementia is reaching epidemic proportions, implying that the analysis of EMRs will provide access to millions of records. While MS can be considered a specialized or “niche disease”, the number of scientific studies and cases in EMRs is too large to explore all this information manually, as is done currently. For this reason, the use of a specific ontology will be very beneficial for clinical and translational research into MS [25]. However, we need to bear in mind that generating more information, even if it is of high quality, does not necessarily mean creating more knowledge. Computational based reasoning systems (clinical decision support systems) are currently being developed, and they may represent the link between automatic information retrieval, research and clinical decision making process [26, 27].

In order to analyze the huge amount of information that has been generated to date, and that can be retrieved with tools such as ontologies, advanced statistical or computational tools must be used in systems medicine approaches, employing neuronal networks, decision trees or network analysis [2, 3]. For example, recent network analysis of co-morbidities has provided significant insights into the relationship between genes, proteins, pathways and chronic diseases ^[^ ^28^, ^29^ ^]^. Similarly, network analysis of drug usage has identified drug combinations that increase or decrease the risk of side effects [30]. These approaches are based on information retrieval from medical databases (Medicare, FDA adverse events database) containing information on millions of patients. However, it usefulness is limited in less prevalent diseases such as MS. Accordingly, the use of a specific ontology for MS may be particularly useful to extract information from EMRs of MS patients, and from clinical and molecular databases, thereby maximizing the identification of new associations between risk factors, molecules, therapies and clinical phenotypes.

One of the great advantages of developing the MS Ontology is that it can be used to extract information from patients with MS contained in the EMRs. However, the analysis of EMRs represents a significant challenge [31, 32]. EMRs contain highly structured information, such as diagnostic codes, but also plain text as in the natural language from physician’s notes. The health systems in different countries also influence the information structure of EMRs. For this reason, there is a tendency to overcode in health systems in which reimbursement is based on coding (e.g. US, Taiwan), whereas there is a tendency to undercode in health systems where reimbursement is based in population coverage (e.g. Europe). In order to avoid the undercoding in the Spanish system, we included ICD-9 codes in our search, but also search terms such as “Multiple Sclerosis” or “demyelinating diseases”. The manual review of the EMRs retrieved revealed that MS Ontology was very efficient in identifying MS cases. Regarding the analysis of natural language from clinical notes using the MS Ontology, we noticed that the search system is sensitive to typographic errors, misspelling, abbreviations and synonyms. For this reason, we updated the MS Ontology dictionary in order to account for such factors, subsequently obtaining greater accuracy. As an example of the mining of EMRs from MS patients, Harvard’s researchers searched EMRs for the estimation of disability (EDSS) and brain atrophy based on the clinical information from MS patients followed in non-specialized MS centers [33]. Current efforts are undergoing in order to make use of EMRs for retrieving information from MS cases, revealing the challenges faced with this approach [34, 35].

Although one can consider that retrieving drug usage in patients with MS is straightforward because these drugs are highly regulated, the true value will come from performing analysis of EMRs from thousand of patients being treated with immunomodulatory drugs in order to understand the patterns of prescription, frequency of adverse-events and to some degree, the efficacy of the therapies (virtual clinical trials). Furthermore, such analyses can be used to understand the role of drugs in disease predisposition at the population level or by using drug prescription as a proxy of co-morbidities (e.g. antibiotics = infection).

In summary, we provide here a new tool for clinical and translational research into MS, which can be used to extract information from the scientific literature, databases and EMRs. Making use of this ontology, we have shown that it is possible to identify new associations between risk factors, molecules, therapies and pathways, and to identify clinical associations with other diseases and therapies. The use of this approach will provide the opportunity to exploit the huge amount of data currently generated by scientific research and clinical care, improving our understanding of MS. However, new computational tools to generate knowledge from this vast amount of information will be required in order to fully benefit from this approach.

## Supporting Information

S1 Methods (DOCX)

S1 Table MS Ontology dictionary. (XLS)

S2 Table Genes identified in GWAS studies. (XLS)

S3 Table Drug usage retrieved from the EMR. (XLS)

S4 Table Comorbidities retrieved from the EMR. (XLS)

S1 Fig Example of the concept extraction of the Pubmed abstracts set using MS Ontology (colors are related with the different levels of the hierarchy of the ontology). (TIFF)

S2 Fig Example of two anonymized medical records with the search of drug (in red) usage and co-morbidities (in blue). (TIFF)
