# Correction: Knowledge Retrieval from PubMed Abstracts and Electronic Medical Records with the Multiple Sclerosis Ontology

**DOI:** 10.1371/journal.pone.0122614

**Published:** 2015-03-13

**Authors:** The PLOS ONE Staff

The eighth author's name is spelled incorrectly. The correct name is: Elena H. Martinez-Lapiscina.
