# Supplementary material for: Knowledge Retrieval from PubMed Abstracts and Electronic Medical Records with the Multiple Sclerosis Ontology
Source: PLoS One. 2015 Feb 9;10(2):e0116718. doi: 10.1371/journal.pone.0116718 (PMC4321837; doi:10.1371/journal.pone.0116718)
Supplement: S1 Methods — (DOCX) [file pone.0116718.s001.docx]

**Supplementary methods**

***MS Ontology***

MSO was constructed in accordance with the ontology-building life cycle [10]. To be compliant with the construction of formal ontologies, we followed the principle criteria of top-level ontologies applying basic formal ontology upper level concepts [11]. The Protege OWL editor (version 4.2) was used as a tool for building MSO in OntologyWeb Language (OWL) format (http://protege.stanford.edu/). Scope and domain coverage of MSO were evaluated by answering three competency questions, which were defined by two experts in clinical/molecular and pharmaceutical MS research. Competency questions are scenarios posed as questions. In other words, they are targets for what the ontology should be able to answer, given sufficient facts (i.e. data) in the knowledgebase.

***Knowledge acquisition and conceptualization***

A first collection of terms and concepts related to MS was generated by scanning various knowledge sources, including review articles, content of online books, standard knowledge bases, encyclopedias, glossaries, and informative online sources and websites. Most of the disease-specific knowledge was acquired by performing web-based searches on resources that focus on various aspects of the disease, such as treatment aspects ([http://www.mayoclinic.com/health/multiplesclerosis/DS00188/ DSECTION=treatments-and-drugs](http://www.mayoclinic.com/health/multiplesclerosis/DS00188/DSECTION=treatments-and-drugs)), neurological perspectives (<http://neurology.health-cares.net>), diagnostic criteria (<http://www.webmd.com/multiplesclerosis/guide/multiple-sclerosis-diagnosing>), research proceedings (<http://www.Omnimedicalsearch.com>), and so forth. Concepts were also extracted from compendiums such as the Multiple sclerosis encyclopedia (<http://www.multsclerosis.org/chooseglossary.html>). Another glossary of terms representing MS was collected from the dictionary of MS [Reference]. Whenever possible, any available hierarchical organization (structure) of the concepts was also extracted along with the concepts themselves. Corresponding definitions and synonyms were also taken into consideration as additional annotation of the concepts.

***Formal representation and concept analysis***

Basic Formal Ontology compliance of MSO as shown in Figure 1 ensures its interoperability with existing and future biomedical ontologies [13]. In order to be able to generate the biological views on root sub-domains of the ontology, axiomatic class definitions were added. By this means, a reasoner can be run to automatically classify other classes as subclasses of these “views”, which are neither a subclass of “occurrent” nor of “continuant”. MSO was tested with regard to formal consistency and absence of cycles using the Fast Classification of Terminologies FaCT++ description logic reasoner [14].

***Synonym enrichment, Terminology analysis and concept enrichment***

Classes were annotated with synonyms, both manually and in an automated way making use of mappings to external ontologies. For this purpose, services provided by the National Center for Biomedical Ontology (NCBO) were used to retrieve synonym information [15]. The dictionary was developed in English and then translated to Spansih and Catalan for the analysis of EHR.

Transformation of the ontology OWL format into a dictionary file was achieved using a Java program that extracts the concept names and the corresponding synonyms from the ontology OWL structure and assigns unique identifiers to each concept which can be stored in form of a dictionary. This dictionary was incorporated into ProMiner, a named entity recognition (NER) software [16]. In a subsequent step, various class concepts were used as keywords for search in PubMed to build a corpus which covers domain information related to topics such as MS biomarkers, brain regions, diagnostic procedure, pharmacology, epidemiology, etiology, genetics, pathogenesis, stages, symptoms, clinical trials, risk factors and many more. From the result list obtained by each concept search, several abstracts were chosen randomly. After compilation of these chosen abstracts, a corpus of 500 abstracts was formed and randomly divided into a training set (250 abstracts), which was used for extracting the terminology manually and building of the dictionary, and an annotation set for the development of the gold standard (250 abstracts). From the latter, a test set of 200 abstracts was selected. In order to create the reference gold standard, suitable annotation guidelines were developed so that the annotator is guided to keep the breadth and depth of the ontology in mind and consider the super class concepts but also their corresponding sub-class concepts as well as their synonyms for annotation.

Using these annotation guidelines, both training and test sets were manually annotated by means of the Knowtator tool (<http://knowtator.sourceforge.net/>). For enrichment purposes (optimizing the dictionary), the training set was analyzed for false-negative entities, which were added to the MSO terminology after individual expert evaluation. Moreover, experts in the field (PV and AS) crosschecked the whole ontology and additional knowledge was incorporated. The test set served as the gold standard as well, because the evaluation process requires the performance comparison between the automatically and manually annotated text from the same set.

To evaluate the quality of the MSO ontology in terms of measuring the boundaries of the knowledge domain that it captures, precision, recall and *F*-score values were calculated. These values were computed based on the longest string match found between automatically annotated terms by ProMiner and the (human) gold standard annotation for each abstract in the selected corpus. The following formulas were used for the computation of recall, precision and *F*-*score* values [17].

Precision= True positives/True positives + False positives

Recall= True positives/True positives + False negatives

*F−score*=2*Precision × recall/Precision +recall

where true positives are the number of entities that were found by ProMiner and that matched the annotation in the gold standard; false positives are the number of entities that were (automatically) annotated by ProMiner but could not be matched to annotations in the (expert annotated) gold standard and false negatives are the number of entities that were not found by ProMiner when compared with the manually (expert) annotated gold standard.

***Visualization of concepts through the text***

To visualize the named entities embedded in the textual body of PubMed abstracts, the transformed XML structure of MSO was integrated into SCAIView [18]. SCAIView is a visualization interface for ProMiner annotations and displays named entities by markup of the text. One key feature of SCAIView is the possibility to perform ontological search in biomedical text using concept hierarchies and synonyms associated with each concept. To adopt MSO in the SCAIView environment, we extracted the inferred “views” on the ontology and transformed the ontology OWL file into XML format containing the “view” taxonomy, labels and synonyms. A ProMiner annotation run with an MSO-derived dictionary generated the meta-information (index) used for searching of MSO terms in SCAIView. Moreover, to reduce the complexity of the hierarchy for better navigation, improve the visualization of the concepts and to increase the specificity, we transferred all the concepts common among general neurodegenerative diseases to a separate hierarchy called neurodegenerative diseases (NDD). This utility along with flattened tree structure of MSO is accessible online at [www.scaiview.com](http://www.scaiview.com).

***MSO structure and contents***

MSO covers a wide range of key concepts and aims to comprehensively represent knowledge specific to MS. Modeled heterogeneity in this semantic framework tries to touch all relevant concepts but the range varies from very general (main ontology classes) to more specific concepts (concepts located at the ontology bottom “leaves”). The main views (root concepts) in MSO covering aspects of MS knowledge domain include: “Clinical features”, “Etiology”, "Models of MS", “Molecular mechanisms on pathways", "Molecular and cellular features", "Social and economic impact of MS". Each of these super-classes has its own subclasses as shown in Figure 2. Subclasses mentioned under “Clinical (features)” cover concepts that have contributed significantly to our understanding of the pathology, diagnosis and possible treatment options. Concepts defined under each subclass tend to be more domain-specific.

“Etiological view” forms the second root in MSO and it covers all aspects that might be responsible for the initiation of MS ranging from genetic factors and environmental influences to morphological changes whose effect varies from individual to individual. Clinical appearance of MS is also marked by anatomical changes as well as cellular and molecular cascades, which together manifest the neuropathological alterations observed in MS.

Also, currently much of the focus of MS research is devoted to preclinical studies that are conducted, typically on animals and has potential to play a vital role in drug discovery and development process. Concepts mapping all aspects of “Non-clinical” studies (*Ex-vivo*, *In-silico*, *In-vitro* and *In-vivo*) have also been incorporated into the "Models of MS" root class of MSO, hence elaborating the knowledge related to animal models and bioassays used to better understand biological processes underlying Multiple sclerosis.

Furthermore, in MSO, the “Molecular and cellular features” and "Molecular mechanisms on pathways" view is designed to cover most entities and biological mechanisms that find a possible role in MS. Studying the behavior of molecules in various cell types can provide insights into the processes that mark the progression of MS. A final root class "Social and economics of MS" is also kept in the ontology having concepts that define socio-economic impact the disease has and also the indirect costs that all these factors induce. The semantic relationships ‘is a’ and ‘has a’ and ‘part of’ were mainly used to define relation types between pairs of concepts.

***MSO evaluation***

The structural features of the ontology reflecting topological and logical properties were measured by means of context-free metrics including depth and breadth (related to the cardinality of paths in a graph), tangledness (related to multi-hierarchical nodes), and fan-outness (related to the dispersion of nodes). Supplementary File 1 shows various parameters, which were considered in the structural evaluation of MSO.

The functional dimension of the ontology reflects the main purpose of that ontology by specifying a set of contextual assumptions about an area of interest. Functional evaluation measures how widely and precisely ontological concepts represent the semantic space for the indicated knowledge domain. The boundary of the knowledge domain addressed by MSO was estimated by calculating its fitness to an existing knowledge repository (i.e. PubMed). Using our state-of-the-art text-mining environment, which takes MSO hierarchical structure and corresponding dictionary as input, we were able to evaluate MSO functionally on the prepared ‘test set’ (see Methods) and report a F-score of 73%. The result of this evaluation shows that the ontology in its current form can capture a wide range of MS concepts in the knowledge domain of MS scattered throughout scientific publications.

The expert panel’s revision of the ontological “view” structure is considered as a genuine evaluation for disease ontologies [19]. Following this, our ontology was manually curated by a team of clinician experts in the field (PV and AS) who added certain clinically relevant concepts to MSO, increasing its pragmatic usability. Two competency questions were defined by expert clinicians and one by an expert from the pharmaceutical industry. The purpose of the competency questions is to evaluate the performance of MSO in semantic searches and its capability to return appropriate answers to the following questions

1. Return references linking brain atrophy and remyelination to MS.
2. Return references linking MOG to antibody-mediated demyelination in MS.
3. Return references linking fingolimod tested as a drug for treatment of relapsing-remitting MS in phase 3 clinical trials

To evaluate the above queries, we manually compared documents returned by MSO using query features of SCAIView with documents returned by PubMed advanced search using the same queries as follow:

***Competency Question 1: Return references linking brain atrophy and Remyelination and multiple sclerosis***

1. PubMed automatic advanced search: (atrophy[Text Word]) AND remyelination[Text Word]) AND multiple sclerosis[MeSH Terms]

PubMed results: 0 documents

2. MSO-SCAIView: atrophy(MSO) + remyelination(MSO)+ Multiple sclerosis(MeSH)

MSO-SCAIView results: 26 documents

PMID: 12815694, 24008938, 16413962 , 15222689, 19546105, 15228754, 18824597, 20737479, 3001568, 12135961, 20373421, 22367995, 22673950, 9845023, 18353554, 19538961, 15882880, 12815708, 11323746, 10467389, 11315199, 12858060, 11408335, 22994884, 14726460, 11193941

3. Expert search (PubMed keywords and manual curation): brain atrophy AND remyelination AND Multiple Sclerosis.

Expert results: 15 documents

PMID: 24239768, 22367995, 20373421, 19546105, 19538961, 18606968, 18353554, 16385018, 15882880, 15222689, 12815694, 11323746, 11043971, 9845023

***Competency Question 2: Return references linking MOG to antibody-mediated demyelination in Multiple sclerosis.***

1. PubMed automatic advance search: (MOG[Text Word] AND antibody-mediated demyelination[Text Word]) AND "multiple sclerosis"[MeSH Terms]

PubMed results: 3 documents

PMID 15259003, 11205153, 11128602

2. MSO-SCAIView search: MOG(MSO) + Antibody mediated demyelination(MSO)+ Multiple sclerosis(MeSH)

MSO-SCAIView results : 9 documents

PMID:15259003, 11205153, 8943561, 11128602, 19422681, 19320002, 17136549, 12503087, 16337942

Expert search (PubMed keywords and manual curation):

Expert results: 9 documents

PMID: 19422681, 19320002, 16337942, 15885306, 15835266, 15259003, 8954853, 8943561, 1991822

***Competency Question 3: Return references linking Fingolimod as a drug for treatment of relapsing-remitting multiple sclerosis to phase 3 clinical trials***

1. PubMed advance search: ((multiple sclerosis, relapsing remitting[MeSH Terms]) AND fingolimod[Text Word]) AND clinical trial[Text Word]) AND phase 3[Text Word]

PubMed results: 1 documents

PMID: 22749258

2. MSO-SCAIView: Multiple Sclerosis Relapsing-Remitting (MSO) AND Phase III (MSO) AND Clinical trial (MSO) AND Fingolimod (MSO)

MSO-SCAIView results: 9 documents

PMID: 18457527, 23410536, 20408749, 22751847, 21158700, 23637535, 17561264, 22284868, 21710707.

Expert search (PubMed keywords and manual curation):

Expert results: 12

PMID: 24494635, 23531349, 23410536, 22749258, 21679286, 21394595, 21151622, 21128693, 19882371, 19812733, 21149809, 20599429

Querying SCAIView with the support of MSO in comparison to querying PubMed using comparable query formulations for all competency questions returns better results in terms of both sensitivity and specificity. These results indicate that MSO-supported information retrieval improves the chances for gaining better coverage with focused results in the same time as compared to naïve PubMed based searches. Also the knowledge gain in terms of concept recognition and enrichment is better when performing ontology based semantic search. To validate this, we used the third competency question and performed the query in SCAIView under two conditions: using the MeSH (Medical subject heading) dictionary (without using MSO) and using MSO in conjunction to the Neurodegenerative diseases terminology [8]. The reason is that the same corpus is indexed behind SCAIView so that the condition for queries remains fairly comparable. We then checked the document retrieval under both conditions and it turned out that the ontology based search using SCAIView (along with all MeSH concepts included as subset), was able to highlight approximately 5 times more entities than was possible using the MeSH dictionary alone. Although ontology based context modeling has been long acknowledged as a key aspect in a wide variety of problem domains, our experiment demonstrates how the combination of ontology-driven semantic search with named entity recognition can define a highly relevant context with high granularity and precision.

**References**

1. Compston, A., & Coles, A. (2008). Multiple sclerosis. *Lancet*, *372*(9648), 1502–17. doi:10.1016/S0140-6736(08)61620-7
2. http://www.statisticbrain.com/multiple-sclerosis-statistics/
3. Rosati, G., 2001.The prevalence of multiple sclerosis in the world: an update. Neuro. Sci.22,117-139.
4. http://www.msif.org/includes/documents/cm_docs/2011/g/global_economic_impact_of_ms.pdf?f=1
5. Bodenreider O. Biomedical ontologies in action: role in knowledge management. Data Integr Decision Supp NIH Public Access 2008;3841:67–79.
6. Ashburner M, Ball CA, Blake JA, Botstein D, Butler H, Cherry JM,et al. Gene ontology: tool for the unification of biology: the Gene Ontology Consortium. Nat Genet 2000;25:25–9.
7. Donnelly K. SNOMED-CT: the advanced terminology and coding system for eHealth. Studies Health Technol informatics 2006;121:279–90.
8. Malhotra A, Younesi E., Gündel M, Heneka M. T., Hofmann-Apitius M. (2013) ADO: a disease ontology representing the domain knowledge specific to Alzheimer’s disease. Alzheimer’s and Dementia, In press.
9. Esposito, Massimo, and Giuseppe De Pietro. "An ontology-based fuzzy decision support system for multiple sclerosis." *Engineering Applications of Artificial Intelligence* 24.8 (2011): 1340-1354.
10. Gómez-Pérez A. et al . Ontological Engineering. Springer, Berlin, 2004.
11. Grenon P, Smith B, Goldberg L.Biodynamic ontology: applying BFO in the biomedical domain. Studies in health technologies and informatics. 2004;102:20-38.[PMID:15853262]
12. Multiple sclerosis dictionary reference
13. Smith B, Kumar A, Bittner T. Basic formal ontology for bioinformatics.2005. Journal of Information System . 1-16.
14. Dmitry Tsarkov and Ian Horrocks . FaCT++ Description Logic Reasoner: System Description. In Proc. of the Int. Joint Conf. on Automated Reasoning (IJCAR).2006; volume 4130 of Lecture Notes in Artificial Intelligence: 292-297.
15. Musen MA, Noy NF, Shah NH, Whetzel PL,Chute CG, Story MA, et al.The National Center for Biomedical Ontology. J Am Med Inform Assoc.2011. 190-195. [PMID:22081220]
16. Hanisch D,Fundel K, Mevissen HT, Zimmer R, Fluck J.ProMiner: rule-based protein and gene entity recognition.BMC Bioinformatics.2005. 6 Suppl 1:S14. [PMID:15960826]
17. Morgan A.A, Lu Z,Wang X, Cohen AM, Fluck J, Ruch P et al. Overview of BioCreative II gene normalization. Genome Biol.2008. 9 (Suppl. 2), S3.[PMID:18834494]
18. Friedrich C.M, et al. @neuLink: a service-oriented application for biomedical knowledge discovery. Proceedings of HealthGrid. 2008. IOS Press, Amsterdam, pp. 165–172.
19. Obrst L. et al. Toward Improved Semantic Interoperability.2007.MITRE New York.,3, 1-19.
20. Jensen, Peter B., Lars J. Jensen, and Søren Brunak. "Mining electronic health records: towards better research applications and clinical care." *Nature Reviews Genetics* 13.6 (2012): 395-405.
21. Li, M. J., Wang, P., Liu, X., Lim, E. L., Wang, Z., Yeager, M., ... & Wang, J. (2012). GWASdb: a database for human genetic variants identified by genome-wide association studies. *Nucleic acids research*, *40*(D1), D1047-D1054.
22. Li, Mulin Jun, et al. "GWASdb: a database for human genetic variants identified by genome-wide association studies." *Nucleic acids research* 40.D1 (2012): D1047-D1054.
23. Mahurkar, S., Moldovan, M., Suppiah, V., & O’Doherty, C. (2013). Identification of Shared Genes and Pathways: A Comparative Study of Multiple Sclerosis Susceptibility, Severity and Response to Interferon Beta Treatment. *PloS one*, *8*(2), e57655
24. Sawcer, Stephen, et al. "Genetic risk and a primary role for cell-mediated immune mechanisms in multiple sclerosis." *Nature* 476.7359 (2011): 214.
25. Duarte NC, Becker SA, Jamshidi N, et al. Global reconstruction of the human metabolic network based on genomic and bibliomic data. Proc Natl Acad Sci USA 2007;104:1777-82.
