# Supplementary figures and images for: Knowledge Retrieval from PubMed Abstracts and Electronic Medical Records with the Multiple Sclerosis Ontology
Source: PLoS One. 2015 Feb 9;10(2):e0116718. doi: 10.1371/journal.pone.0116718 (PMC4321837; doi:10.1371/journal.pone.0116718)

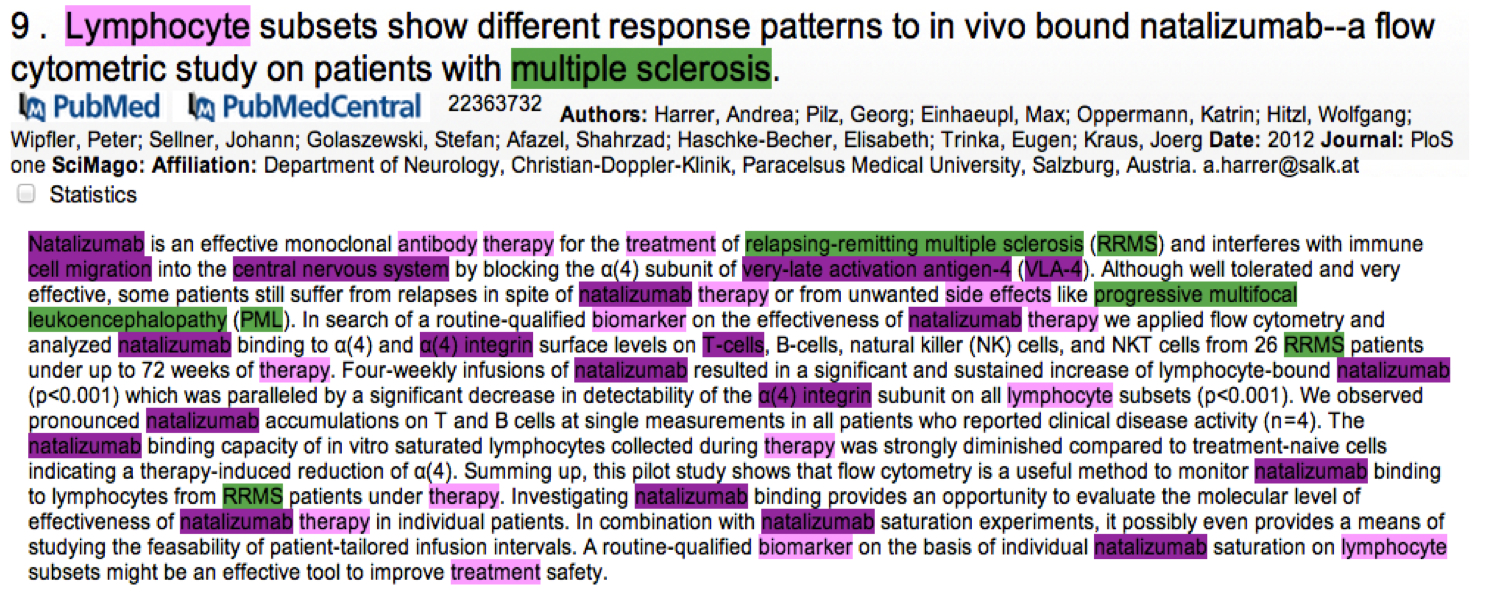

Supplement: S1 Fig — (TIFF) [file pone.0116718.s006.tiff]

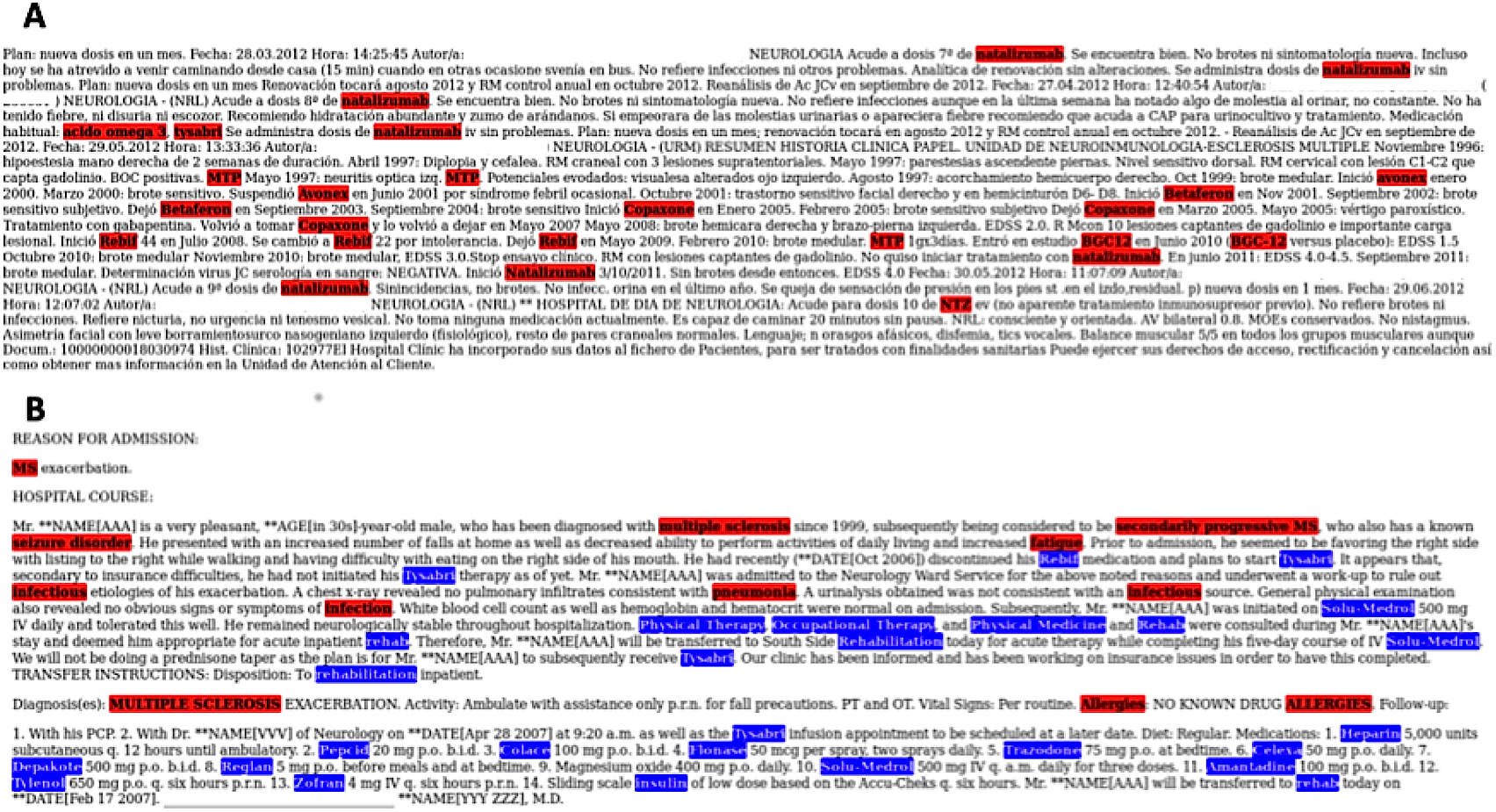

Supplement: S2 Fig — (TIFF) [file pone.0116718.s007.tiff]
